# The antioxidant defense capacities and histological alterations in the livers and gills of two fish species, *Oreochromis niloticus* and *Clarias gariepinus*, as indicative signs of the Batts drain pollution

**DOI:** 10.1007/s11356-022-20804-y

**Published:** 2022-05-23

**Authors:** Moussa Attia Moussa, Hanan Ramadan H. Mohamed, Amr Adel Abdel-Khalek

**Affiliations:** Zoology Department, Faculty of Science, Cairo University, Cairo, Egypt

**Keywords:** Metal pollution, Antioxidant responses, Histological alterations

## Abstract

The impacts of the Batts drain on two chronically exposed fish (*O. niloticus* and *C. gariepinus*) were assessed using multiple biomarkers. Concentrations of metals in water and sediments (Cu, Zn, Fe, Cd, Pb, and Al) showed significant elevations near the Batts discharges (site 2) compared to the reference site (site 1). The liver and gills of fish collected from site 2 showed marked elevations in the catalase, superoxide dismutase, glutathione peroxidase, and thiobarbituric acid reactive substance levels. In addition, significant reductions in glutathione-reduced contents were also recorded. Tissue and species-specific antioxidant responses were associated with excessive generations of reactive oxygen species, which were visualized fluorescently. Various histological alterations were observed in the gills and livers of both species. These alterations varied between compensatory responses (ex: epithelial thickening and lifting) and irreversible damage (ex: necrotic degeneration). Based on the level of lipid peroxidation and the frequency of histopathological modifications, *O. niloticus* demonstrated greater resistance to the same level of pollution than *C. gariepinus*. Using integrated biomarkers to evaluate the real impacts of untreated discharges of the Batts drain is applied for the first time on the selected fish species at the studied sites.

## Introduction

Metal pollution of aquatic bodies, especially closed lakes, is a global environmental problem. Despite the existing environmental conservation laws, the unregulated waste disposal associated with ineffective water management has resulted in the deterioration of many Egyptian lakes (Abdel-Khalek et al. 2020). Lake Qaroun is one of the most affected lakes by anthropogenic activities. It is located in the Fayoum depression on the edge of Egypt’s western desert, about 80 km from Cairo’s southwest, and covers about 226 km^2^. It is a closed basin with a 40 km length, 5.7 km width, and 4.2 average depth. The Batts is a significant drain that pours its drainage into the lake. This drain collects agricultural discharges (the main cause of metal pollution) from the Fayoum province’s eastern and northeastern regions and directs them to lake Qaroun (Zaghloul et al. 2011). Therefore, lake Qaroun and its biota are suffering from the high pollution loads that come from the Batts drain. A massive amount of metals has accumulated in the aquatic ecosystem as a result of the massive release of untreated discharges, causing several toxicological problems in the exposed organisms (Naz et al. 2021). Aqueous metals are among the most destructive contaminants due to their high bioaccumulation efficiency, persistence, and ability to interact with numerous biological components (Rajeshkumar et al. 2018; Sauliutė et al. 2020). Metal ions can excessively induce reactive oxygen species (ROS) generation through different metal-related reactions. For example, transition metal ions can change the valence state; therefore, they support the Fenton reactions by producing hydroxyl radicals (Temiz and Kargın 2022). Hence, redox-active metals (for example, Cu and Fe) might disrupt essential non-enzymatic and enzymatic antioxidant components and mediate the overproduction of ROS (Hermenean et al. 2015). Excessive ROS production with a defective scavenger capacity of the antioxidant defense system may cause oxidative stress and high lipid peroxidation levels (Massoud et al. 2021). Catalase (CAT), superoxide dismutase (SOD), glutathione peroxidases (GPx), and reduced glutathione (GSH) are part of the cellular antioxidant defense system against the adverse effects of ROS. Relying on the classical chemical analyses of different aquatic pollutants in the environmental components only (ex: water, sediment) cannot determine the real toxicological impacts of these pollutants on various organisms. Therefore, the cellular antioxidant battery and the level of ROS production are recommended as valuable biomarkers during environmental studies (Turan et al. 2020). Moreover, the cell membranes (lipid-rich cellular components) are vulnerable to being targeted by ROS, so thiobarbituric acid reactive substances (TBARS; one of the breakdown products of the lipid peroxidation process) can be used as a good indicator for oxidative damage (Hedayati et al. 2021). Using integrated biomarkers is an effective tool for monitoring environmental stresses and reveals critical information concerning the toxicological effects on chronically exposed living organisms that chemical approaches cannot detect (Greani et al. 2017). Among the most indicative biomarkers, histopathological changes can provide early alarms for chronic stressors on various tissues and the overall health status of fish (Abdel-Khalek et al. 2016). For example, *Oreochromis niloticus* (Nile tilapia) and *Clarias gariepinus* (Catfish) are suitable animal models for ecotoxicological field studies due to their high sensitivity and resistance to various aquatic contaminants (Turan et al. 2020; Abu Shnaf et al. 2021). Therefore, the present research aimed to (1) impact analysis of the Batts drain discharges on the health profile of *O. niloticus* and *C. gariepinus* involving an integrative approach of oxidative stress and histological biomarkers and (2) compare the different responses of two fish species facing the same pollution level. Applying those integrated biomarkers for the first time at the studied sites can assess the real impacts of the Batts drain on the selected fish species, in addition identify the most susceptible and resistant fish species based on their health profile.

## Materials and methods

### Sampling sites

**Fig. 1:**
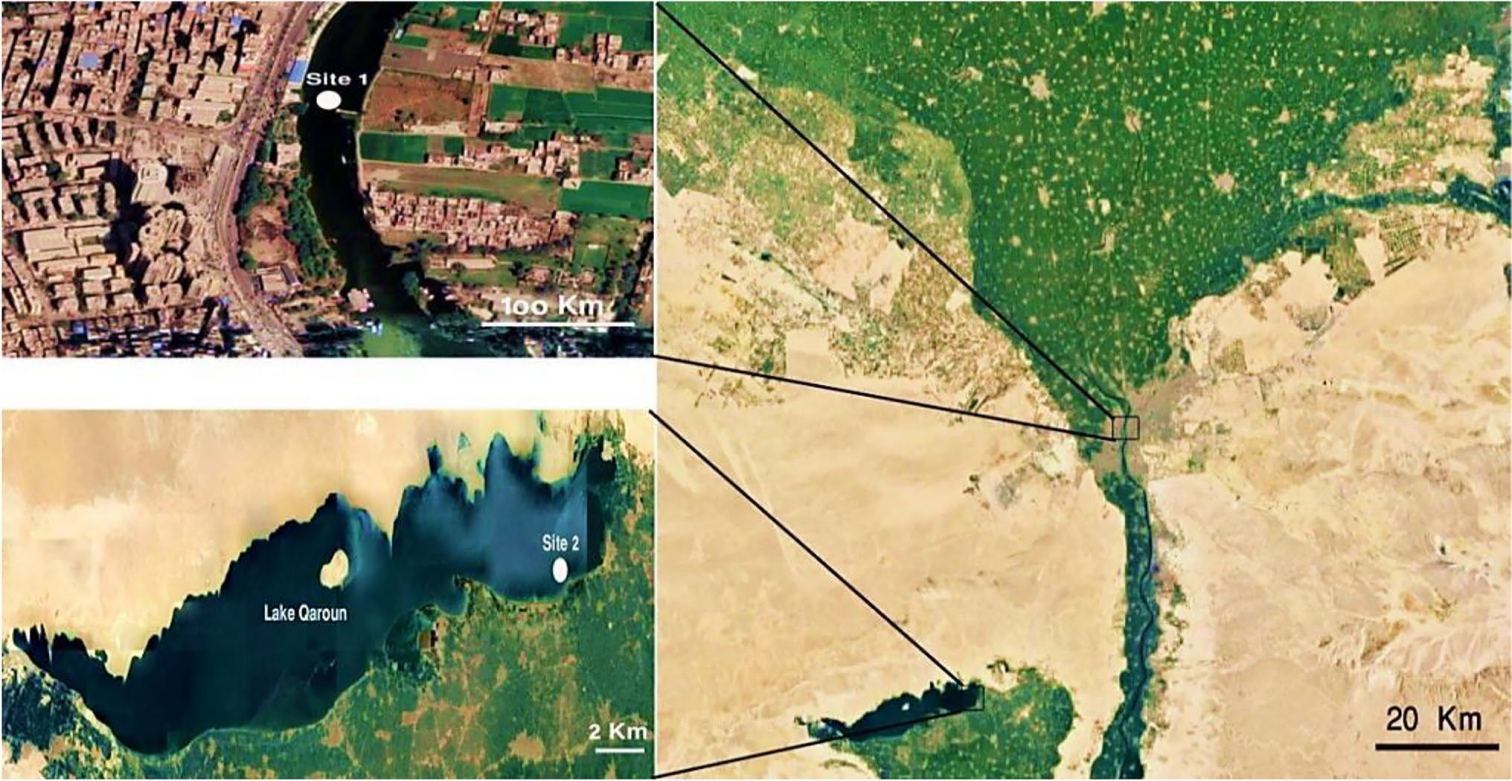
Map of the sampling sites

The sampling sites were as follows: Site 1 (which represents the reference site) was on the River Nile, south of Giza governorate, Egypt. This site was chosen because of its distance from any source of pollution and effluent discharge. GPS: 30° 00′ 02.034″ N and 31° 12′ 55.7532″ E. Site 2 (at the inlet of the Batts drain): The majority of human activities are nearby the eastern section of lake Qaroun and the largest number of agricultural drains extends from the Batts drain. The Batts drain discharges its contents directly into lake Qaroun; therefore, site 2 was selected close to the discharge point of this drain. GPS: 29° 28′58.98″ N and 30° 49′08.02″ E. (Fig. 1).

### Water, sediment, and fish collection

Water sampling was performed using standard procedures by APHA (2017). Water samples (*n* = 4 per site) were collected from the studied sites in glass containers. For metal analysis, concentrated hydrochloric acid was added to decrease the pH to < 2 of the obtained samples to prevent microbial reactions. Metals in sediment are inert, but they can slowly leak into the water column in response to various events that could endanger ecosystems. To safeguard the aquatic environment, accurate evaluation of metal contamination in sediments near water is required to assess and identify the impact of natural and/or anthropogenic resources. The sediment samples (*n* = 4 per site) were collected in polyvinyl chloride (PVC) bottles from each site at 20 cm depth, then locked and stored at 4 °C as detailed by Cabrera et al. (1992).

The two selected fish species, *Oreochromis niloticus* and *Clarias gariepinus*, were caught during the summer season of 2019 (period of maximum evaporation rate and maximum pollutant concentrations), with expert fishermen’s help. The 36 adult male fish of both species (9 fish/species/site) with mean body weights of 195.51 ± 7.46 and 353.67 ± 24.4 g and average total lengths of 19.95 ± 0.36 and 37.91 ± 1.02 cm for *O. niloticus* and *C. gariepinus*, respectively, were obtained from the studied sites. Fish were transferred in large plastic containers with excellent aeration conditions to the ecology laboratory of the Zoology Department, Faculty of Science, Cairo University, using portable oxygen pumps. The dissection of fish was done after decapitation, and the gills and hepatic tissues were isolated for further studies. The Institutional Committee on Animal Care and Use (IACUC) of Cairo University, Faculty of Science, Cairo, Egypt, reviewed and approved this study with the accreditation number CU-I-F-39–19.

### Metal analysis

The concentrations of Cu, Zn, Fe, Pb, Cd, and Al metals were determined in water and sediment using Inductively Coupled Argon Plasma, ICAP 6500 Duo Thermo Scientific, England. An amount of 1000 mg/L multi-element certified standard solution (Merck, Germany) was used as a stock solution for instrument standardization. The detection limits are shown in Table 1.

**Table 1:** The limits of detection for all studied metals

Elements	The detection limits in ppm
Cu	0.002
Al	0.001
Cd	0.0005
Zn, Pb, and Fe	0.005

Acid digestion of sediment samples was conducted according to the procedure mentioned by Neugebauer et al. (2000). The samples were dried at 80 °C in an oven for 8 h to dry out entirely. The samples were then digested with concentrated hydrochloric acid. The mixtures were carefully stirred and placed on a heat plate; then, the temperature was gradually increased to 100 °C until the appearance of a clear solution. The resulting solutions were transported to a 25-mL volumetric flask and diluted to a known volume with de-ionized water. The studied metal concentrations were expressed as mg/L in water and as mg/kg dry weight in sediments.

### Quality assurance and quality control procedures

To correct background absorption, blank samples (prepared in the same way as samples but without the sample) were prepared along with each sample set. The analysis procedure was validated using standard reference material (Lake Superior fish 1946 NIST, National Institute of Standards and Technology, USA), with metal recovery ranges ranging from 94 to 105%. A standard solution with a known concentration of each metal was used during the measuring process to verify the accuracy of the measurement.

### Antioxidant biomarkers

Tissue homogenates for gills and livers were prepared (0.5 g tissue in 2.5 mL of cold specific buffer for each antioxidant biomarker) and centrifuged at 1073 g for 10 min in a cooling centrifuge (4 °C). All antioxidant biomarkers were measured using biodiagnostic kits from Biodiagnostic Dokki, Giza, Egypt. The activities of CAT (CA 25 17) enzymes (U/g proteins) were estimated based on terminating the reaction between CAT and the specified H_2_O_2_ volume (after a certain time) with a catalase inhibitor as detailed by Aebi (1984). The specific buffer used in the homogenization was 50 mM potassium phosphate, pH 7.4, 1 mM EDTA, and 1 mL/L Triton X100. As described by Nishikimi et al. (1972), SOD (SD 25 21) can inhibit the phenazine methosulphate–mediated reduction of nitroblue tetrazolium dye. Therefore, SOD activities (U/g proteins) were measured according to the inhibition rate of the previous reaction. The SOD homogenizing solution was 100 mM potassium phosphate, pH 7.0, containing 2 mM EDTA. In the presence of glutathione reductase, the GPx enzyme (GP 2524) can convert organic peroxide to oxidized glutathione, which is then recycled to its reduced state. Based on the reducing properties of the GPx enzyme, the activities of GPx (U/g proteins) were colorimetrically determined as shown by Paglia and Valentine (1967). The buffer constituents were 50 mM phosphate buffer, pH 7.0, containing 5 mM EDTA and 1 mM 2-mercaptoethanol. Utilizing the ability of GSH (GR 25 11) to reduce 5, 5′-dithiobis 2-nitrobenzoic acid into a yellow-colored compound, GSH could be assessed (mmol/g protein) colorimetrically at 405 nm as provided by Beutler et al. (1963). The homogenizing buffer components were 50 mM potassium phosphate, pH 7.5, and 1 mM EDTA. The concentration of TBARS (a byproduct of lipid peroxidation) was measured (nmole/g tissue) according to the method of Ohkawa et al. (1979), in which the TBARS (MD 25 29) reacts with thiobarbituric acid, generating a colored end-product. The color intensity at 534 nm was equivalent to TBARS concentrations. The homogenizing buffer was 50 mM potassium phosphate, pH 7.5.

### Visualization of generated ROS

As Wang and Joseph (1999) and Siddiqui et al. (2010) described, ROS generation was determined in the gills and livers of both selected species collected from the studied sites. The method relies on the passive passing of 2,7-dichlorofluorescin diacetate (DCFH-DA), which interacts with ROS to produce the highly fluorescent chemical dichlorofluorescein compound (DCF). After homogenizing the studied tissue samples, DCFH-DA (20 mM) was added to the cell suspension, and the samples were kept in the dark for 30 min before being visualized and photographed with a fluorescence microscope (Optika B 353LD2 LED trinocular fluorescence microscope) at excitation and emission wavelengths of 485 nm and 528 nm, respectively, and × 20 magnification. For ROS quantification, each cell in each image was analyzed using Image J software (version 1.50, USA) and the data are represented as corrected total cell fluorescence (CTCF). CTCF = integrated density − (area of selected cell × mean fluorescence of background).

### Histopathological alterations

The gills and liver tissues were isolated and washed in a physiological saline solution (0.9% NaCl) to remove excess blood or any debris. The washed tissues were conserved in a 10% formalin solution. According to Bernet et al. (1999), the preserved tissues were processed in graded series of ethanol, paraffin sectioned at 4 μm, and then stained using Hematoxylin and Eosin and finally photographed by light microscopy. Nine specimens/tissue/species/site were used in the histological study, and the percentage of common alterations was calculated (*n* = 18; 2 slides of different 9 fish). After the examination of all recorded alterations in all slides (18/tissue/species/site), the percentage of appearance for each alteration was calculated as shown:

$$\mathrm{\% of appearance}=\frac{\mathrm{The number of slides in which the alteration recorded}}{\mathrm{total number of slides }(18)}\times 100$$

### Statistical analysis

All data were expressed as mean ± SE. The raw data were normally distributed as determined by the Shapiro–Wilk and Kolmogorov–Smirnov tests, as well as homogenous as determined by Levene’s test. Data were statistically analyzed (*P* < 0.05) with Student’s *t*-test, ANOVA test, and Duncan’s test to evaluate differences among different fish and sites. The statistical analysis was conducted using Statistical Processor Systems Support, SPSS software, version 25.0, IBM, Chicago, IL, USA.

## Results

### Concentrations of metals in water and sediments

The concentrations of metals in water and sediments collected from the studied sites are presented in Table 2. A significant (*P* < 0.05) increase in all measured metals was observed in samples from Site 2 (except for Cu concentration in water) compared to the reference site. Metal concentrations in water and sediment samples from site 1 were within the permissible concentrations based on international and national guideline values for metal concentrations in aquatic bodies (MacDonald et al. 2000; MWRI 2013). The concentration of all metals in water samples at site 2 exceeded the guideline values except for copper metal. The concentrations of metals in sediment samples of site 2 were less than the guideline values except for Cu and Cd metals.

**Table 2:** Aqueous (mg/l) and sedimentary (mg/kg dry wt.) metal concentrations in the studied sites (*n* = 4)

		Site 1 (Reference site)	Site 2 (The Batts site)	Guideline values (MacDonald et al. (2000); MWRI (2013))
Copper	Water	0.289 ± 0.0825^a^	0.2654 ± 0.141^a^	0.5 mg/l
	Sediment	10.74 ± 1.08^b^	37.32 ± 1.51^a^	31.6 mg/kg
Zinc	Water	1.32 ± 0.22^b^	26.85 ± 3.02^a^	2 mg/l
	Sediment	26.25 ± 2.19^b^	100.19 ± 14.69^a^	121 mg/kg
Iron	Water	1.94 ± 0.166^b^	56.59 ± 5.21^a^	3 mg/l
	Sediment	2579.15 ± 141.69^b^	6500.12 ± 182.61^a^	20.000 mg/kg
Cadmium	Water	0.0019 ± 0.0002^b^	0.0049 ± 0.00019^a^	0.003 mg/l
	Sediment	0.6733 ± 0.079^b^	4.063 ± 0.796^a^	0.99 mg/kg
Lead	Water	0.029 ± 0.0068^b^	5.152 ± 0.465^a^	0.1 mg/l
	Sediment	4.806 ± 0.356^b^	17.99 ± 3.312^a^	35.8 mg/kg
Aluminum	Water	0.055 ± 0.005^b^	4.35 ± 0.296^a^	NA
	Sediment	4.14 ± 0.747^b^	15.62 ± 2.58^a^	NA

- Means with the same letter in the same row for each parameter are not significantly different (*P* < 0.05); otherwise, they do

- *NA*, not available

### Enzymatic and non-enzymatic antioxidant biomarkers

The studied enzymatic and non-enzymatic antioxidant biomarkers in the liver of *O. niloticus* and *C. gariepinus* collected from the studied sites are represented in Table 3. Compared to the reference fish, the gills of both studied species showed elevated enzymatic activities of CAT, SOD, and GPx in addition to increased TBARS concentrations. Sharp decreases in GSH levels were also recorded in both species collected from the polluted site (the Batts). The maximum lipid peroxidation rate (as indicated by TBARS level) is recorded in *C. gariepinus* of site 2. As shown in Table 4, there are significant elevations in CAT and GPx (maximally in *O. niloticus* at site 2) and SOD (maximally in *C. gariepinus* at site 2) compared to the reference fish, while GSH levels were sharply decreased in both fish species of site 2. The enzymatic and non-enzymatic antioxidant biomarkers in the liver showed the same trend as in gills but with lower TBARS concentrations indicating more effective antioxidant responses and less lipid peroxidation rate.

**Table 3:** The antioxidant biomarkers in the gills of *O. niloticus* and *C. gariepinus* collected from the studied sites

	**Site 1** **(Reference site)**		**Site 2** **(The Batts site)**	
	*O. niloticus*	*C. gariepinus*	*O. niloticus*	*C. gariepinus*
CAT (U/g proteins)	545 ± 45.02^b^	344 ± 20.44^c^	826 ± 90.78^a^	482 ± 60.9^bc^
SOD (U/g proteins)	166 ± 25.95^c^	139 ± 22.9^c^	2455 ± 162.85^a^	1602 ± 222.2^b^
GPx (U/g proteins)	205 ± 10.7^c^	199 ± 30.05^c^	337 ± 3.18^b^	455 ± 63.09^a^
GSH (mmol/g protein)	3.38 ± 0.23^a^	3.1 ± 0.06^a^	2.36 ± 0.15^b^	1.6 ± 0.12^c^
TBARS (nmole/g tissue)	44.3 ± 1.2^c^	46.15 ± 2.05^c^	60.56 ± 0.64^b^	67.14 ± 0.7^a^

- Data are represented as means of six samples per species in each site ± SE

- The capital letters represent the Duncan’s test (*P* < 0.05) between the different species of the different sites: means with the same letter in the same row for each biomarker are not significantly different; otherwise, they do

**Table 4:** The antioxidant biomarkers in the liver of *O. niloticus* and *C. gariepinus* collected from the studied sites

	**Site 1** **(Reference site)**		**Site 2** **(The Batts site)**	
	*O. niloticus*	*C. gariepinus*	*O. niloticus*	*C. gariepinus*
CAT (U/g proteins)	768 ± 11.68^b^	500 ± 24.46^c^	1372 ± 119.76^a^	728 ± 41.43^b^
SOD (U/g proteins)	630 ± 30.49^b^	781 ± 113.92^b^	880 ± 93.29^b^	1136 ± 65.93^a^
GPx (U/g proteins)	343 ± 63.9^b^	208 ± 15.13^c^	573 ± 13.7^a^	346 ± 16.79^b^
GSH (mmol/g protein)	5.9 ± 0.18^b^	8.5 ± 0.09^a^	4.6 ± 0.42^c^	5.2 ± 0.32^bc^
TBARS (nmole/g tissue)	29.46 ± 3.05^c^	30.51 ± 0.52^c^	44.5 ± 3.55^b^	57.12 ± 2.47^a^

- Data are represented as means of six samples per species in each site ± SE

- The capital letters represent Duncan’s test (*P* < 0.05) between the different species of the different sites: means with the same letter in the same row for each biomarker are not significantly different; otherwise, they do

### Visualization of generated ROS in gills and livers of studied species

**Fig. 2:**
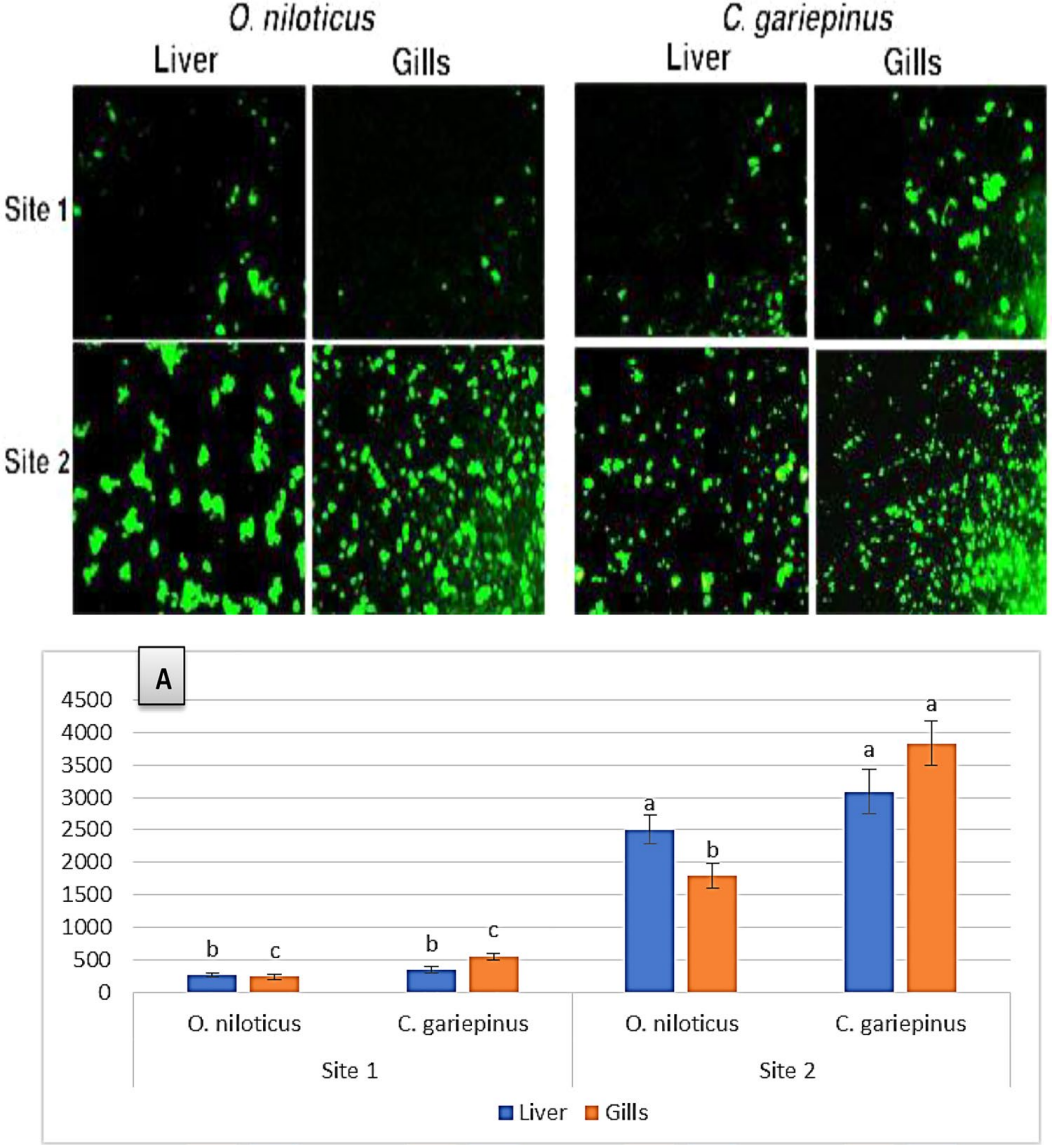
The photographic images of ROS generation in the liver and gills of *O. niloticus* and *C. gariepinus* collected from the studied sites. Graph (A) shows a significant (*P* < 0.05) induction in ROS exemplified as corrected total cell fluorescence (CTCF) in the liver and gills of the studied fish (columns with the same letter in for each tissue are not significantly different (*P* < 0.05); otherwise, they do)

In Fig. 2, the results of ROS generation in gills and livers are represented. The microscopic fluorescence images revealed excessive production of intracellular ROS (indicated by the high intensity of fluorescence) in the gills and livers of both studied species inhabiting site 2.

### Histopathological alterations

**Fig. 3:**
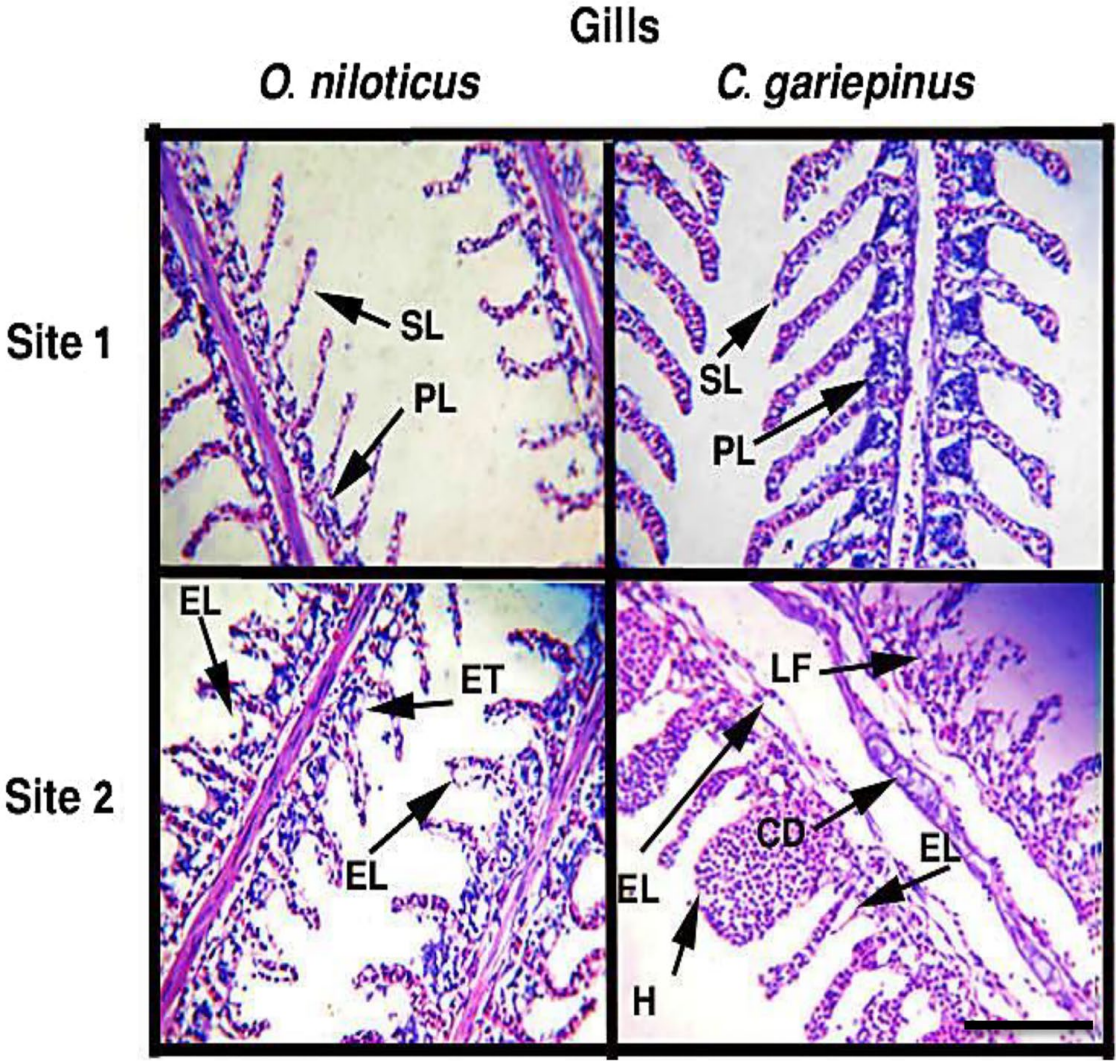
The histopathological alterations in the gills of *O. niloticus* and *C. gariepinus* collected from the studied sites. PL, primary lamella; SL, secondary lamella; ET, epithelial thickening; EL, epithelial lifting; H, hyperplasia; LF, lamellae fusion; CD, cartilaginous deformation. Scale bar = 100 μm

**Fig. 4:**
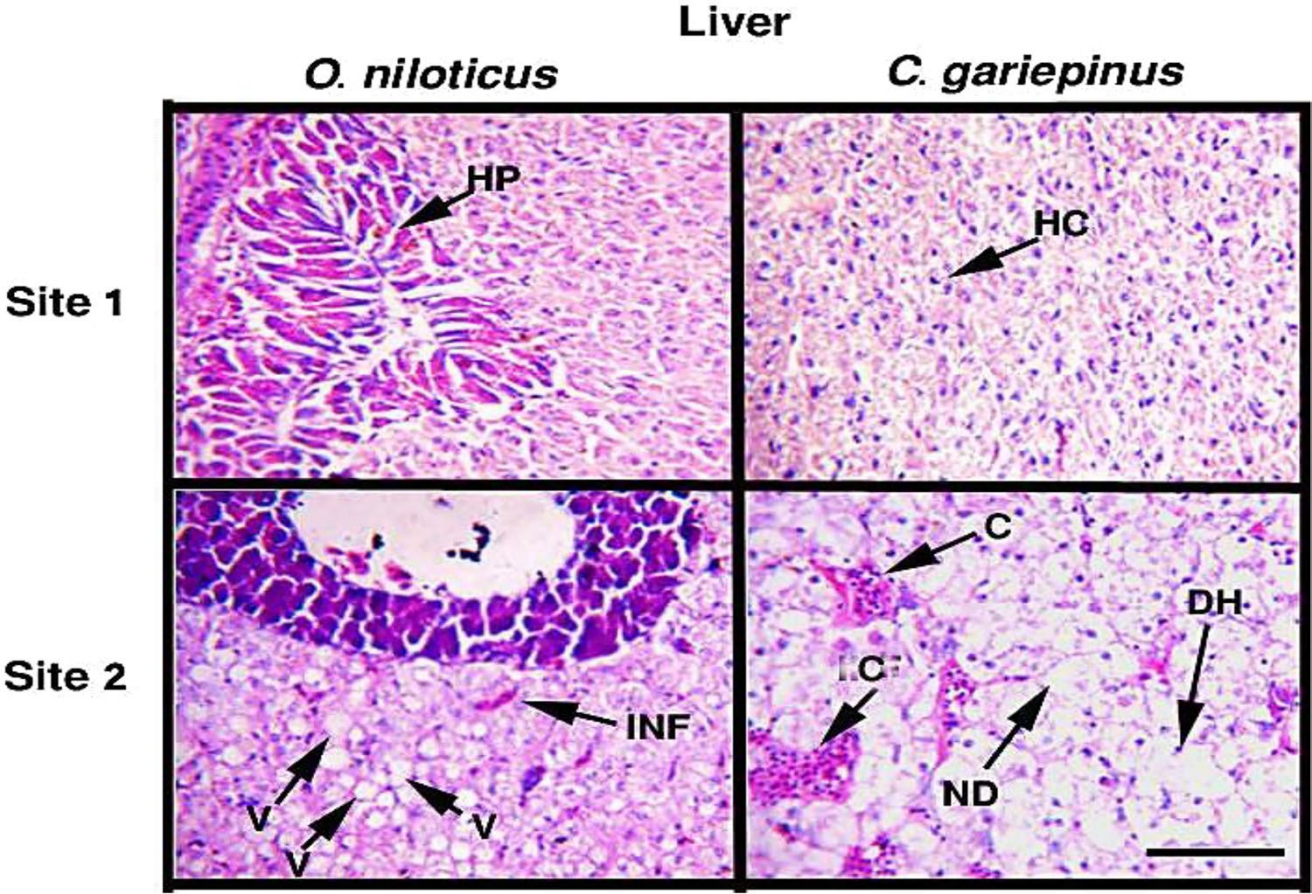
The histopathological alterations in the livers of *O. niloticus* and *C. gariepinus* collected from the studied sites. HP, hepatopancreatic tissue; INF, infiltration of red blood cells; V, vacuolation; HC, hepatocytes; ND, necrotic damages; C, congestion; DH, disarranged hepatocytes. Scale bar = 100 μm

Figure 3 shows the histopathological alterations in the gill tissues of both species under study. The structural deformities in the studied tissues were widely observed in specimens collected from site 2. Several alterations such as epithelial lifting, fusion in secondary lamellae, epithelial thickening, hyperplasia, and cartilaginous deformation were recorded in gills. The observed histopathological changes in the liver (Fig. 4) were disorganized hepatocytes, infiltration of red blood cells, vacuolization in hepatocytes, necrotic damage, congestion in hepatopancreatic tissue, and congestion in hepatocytes. Based on the frequency of deformities as shown in Table 5, the tissues collected from site 1 showed normal histological structures and regular cellular arrangements with less observable structural damage compared to the tissues from site 2.

**Table 5:** The % of the recorded histopathological modifications in gills and livers of *O. niloticus* and *C. gariepinus* from the studied sites. *N* = 18 specimens/site (2 slides from 9 fish in each site)

Recorded alterations	Site 1 (Reference site)		Site 2 (The Batts site)	
	*O. niloticus*	*C. gariepinus*	*O. niloticus*	*C. gariepinus*
**Gills**				
Epithelial lifting (at the tips or bases)	4/18 (22.2%)	5/18 (27.7%)	18/18 (100%)	18/18 (100%)
Fusion in secondary lamellae	2/18 (11.1%)	3/18(16.7%)	16/18 (88.8%)	18/18 (100%)
Epithelial thickening	2/18 (11.1%)	4/18(22.2%)	16/18 (88.8%)	17/18 (94.4%)
Hyperplasia	2/18 (11.1%)	2/18(11.1%)	11/18 (61.1%)	15/18 (83.3%)
Cartilagenous deformation	3/18 (16.7%)	2/18(11.1%)	9/18 (50%)	13/18 (72.2%)
**Total recorded alterations**	**13/90 (14.4%)**	**16/90 (17.7%)**	**70/90 (77.7%)**	**81/90 (90%)**
**Liver**				
Disarranged hepatocytes	2/18 (11.1%)	2/18 (11.1%)	12/18 (66.7%)	14/18 (77.7%)
Infiltration of red blood cells	1/18 (5.6%)	3/18(16.7%)	11/18 (61.1%)	17/18 (94.4%)
Vacuolization	2/18 (11.1%)	3/18(16.7%)	18/18 (100%)	18/18 (100%)
Necrotic damages	0/18 (0%)	1/18 (5.6%)	11/18 (61.1%)	13/18 (72.2%)
Congestion in hepatopancreatic tissue	0/18 (0%)	0/18 (0%)	5/18 (27.7%)	0/18 (0%)
Congestion in hepatocytes	1/18 (5.6%)	1/18 (5.6%)	11/18 (61.1%)	17/18 (94.4%)
**Total recorded alterations**	**6/108 (5.6%)**	**10/108 (9.3%)**	**68/108 (63%)**	**79/108 (73.2%)**

## Discussion

The dense anthropogenic activities nearby lake Qaroun and the massive effluents through the Batts drain have badly affected the optimal inhabiting conditions of many fish species. These untreated discharges not only alter the chemical equilibrium of the water but also deteriorate the health status of the chronically exposed fish. Lake Qaroun was designated as one of the hotspots of metal contamination in El-Fayoum province (Abdel-Khalek et al. 2020). Metal bioaccumulation has been linked to the emission of agricultural and industrial effluents, as well as waste dumping in aquatic media, which can harm the aquatic environment. The majority of studied metals in water and sediment samples at site 2 exceeded the safe values recommended by the Ministry of Water Resources and Irrigation (Egypt) for non-freshwater bodies (MWRI 2013). This site is impacted by the Batts drain (the largest drain at lake Qaroun), which receives agricultural drainage water from the eastern side of El-Fayoum valley, as well as several anthropogenic activities. These findings were in line with those of Khalil et al. (2017), who found that the eastern section of lake Qaroun had higher metal pollution levels than the western side due to the impact of the Batts drainage and numerous anthropogenic activities in this area. Individual metal toxicity assessments may not provide a realistic environmental scenario because science metals in combination exhibit additive toxicity when compared to their individual effects (Javed et al. 2017). Thus, studying internal toxicological responses of suitable bio-indicators provides more accurate interactive hazardous effects compared to external metal speciation analysis. In this context, the present study evaluated the impact of untreated water discharges as a continuous source of metal pollution on multiple biomarker responses. Exposure to high metal concentrations promotes ROS overproduction such as superoxide anion radical, hydroxyl radical, and hydrogen peroxide (H_2_O_2_) through several mechanisms such as Fenton- and Haber–Weiss-type reactions (Saglam et al. 2014). The generated ROS leads to significant mobility of antioxidant components to mitigate the harmful effects of these free radicals. Because the liver is the primary pollutant detoxification/biotransformation tissue and the gills are external tissues that are constantly exposed to water, both tissues are used to assess oxidative stress and fish ability to scavenge excess ROS (Ogunwole et al. 2021). The complementary reactions of SOD/CAT enzymes are classified as the earliest defense mechanisms against free radicals. The role of both enzymes is to convert superoxide radicals to oxygen (O_2_) and H_2_O_2_, and finally into inert water molecules (Yousefi et al. 2021). The activation of both co-working enzymes indicates a massive generation of ROS and hence excessive production of H_2_O_2_. These results are in agreement with Turan et al. (2020) who demonstrated a positive relationship between CAT-SOD activations in *C. gariepinus* and aqueous metal exposure from the polluted Orontes River. Kumar et al. (2021) suggested CAT and SOD as trustworthy biomarkers for biomonitoring of metal pollution in marine ecosystems using thirty different fish species. GPx is an important enzyme for detoxifying H_2_O_2_ and organic peroxides (Temiz and Kargın 2022). The enhancement of SOD and CAT responses was combined with a significant elevation in the GPx activities, revealing that the SOD-CAT defense mechanism could not scavenge the excessive ROS and that the participation of GPx was necessary. Continuous and chronic exposure to metal pollution increases the conjugation rate between metals and GSH (through its thiolate sulfur atom), forming GS–metal complexes (Javed et al. 2016). The strong affinity of SH residues in GSH for metals explains the recorded reduction in GSH content (abundant thiol molecule) of fish collected from the polluted site. The induction of several enzymatic and non-enzymatic antioxidant defense components was unable to avoid lipid peroxidation as signposted by elevated TBARS levels in the liver and gills of site 2. The ineffective antioxidant capacities after chronic exposure to mixed metal pollution were in agreement with Arojojoye et al. (2018), who reported altered antioxidant responses in the liver and muscle of *Clarias gariepinus* collected from the Igbokoda River in South-Western Nigeria. Based on the overall induction of antioxidant components and the level of TBARS (a byproduct of lipid peroxidation), *O. niloticus* showed more significant potential and flexibility to resist the continual flux of superoxide radicals compared to *C. gariepinus* facing the same pollution level. ROS, when present at a proper physiological level, has key functions in regular cell functions, such as antigen fighting, regulating various intercellular signals, and allowing appropriate maturation in reproductive systems (Sinenko et al. 2021). However, when ROS are particularly abundant and exceed the antioxidant coping capacities, oxidative stress can lead to cellular dysfunction via peroxidation of lipids, damage to proteins, and DNA (Ibrahim et al. 2021). Oxidative stress is linked to several pathologies, including histopathological damage, because of its harmful influence on cells (Alchalabi et al. 2016). The most common histopathological alterations in gills were epithelial thickening of primary lamellae, epithelial lifting at the bases and tips of secondary lamellae, severe hyperplasia with ballooning swelling in secondary lamellae, and secondary lamellar fusion. The adaptive anatomical changes such as gill hyperplasia and detached lamellar epithelium may increase the depth of the epithelial cell layer, providing an effective barrier between the exterior and interior environments (Marinović et al. 2021). Furthermore, the reported telangiectasis (ballooning swelling) might be used as ion storage to accumulate metals from water and enhance the adhesion between lamellae to develop an anatomical barrier against external pollutants (Santos et al. 2021). If several telangiectatic lamellae are present, the respiratory function may be compromised, and if the fish is further stressed, a severe reduction in oxygen concentration may occur (Marinović et al. 2021). Blood flow increases in newly established conditions of reduced oxygen concentration, increasing the level of circulatory disturbances. The most pronounced circulatory alteration, according to Rašković et al. (2013), was hyperemia, which represents an increased blood supply due to a disruption in gas exchange and telangiectasis which represents reversible swollen blood vessels in the secondary gill lamellae. The fusion of lamellae may reduce the surface area exposed to the contaminants of the Batts effluents. The vacuolization of hepatocytes was reported in *O. niloticus* exposed to metal pollution as a mark of abnormal accumulation of fat in the cytoplasm, protein breakdown, and metabolic malfunction (Massoud et al. 2021). The appearance of hepatic venules occupied by red blood cells indicates a consequence of the loss of cellular membrane integrity. These changes are often linked to a chronic and progressive necrotic state (Mahboob et al. 2020). The severe histological damage in gills and liver tissues indicates the direct injurious impacts of metals that are in continuous contact with those tissues during ion exchanges and the detoxification processes. Furthermore, oxidative stress and massive ROS accumulation have been linked to a variety of cellular and histological changes in fish (Javed et al. 2017).

## Conclusion

These integrated biomarkers were applied for the first time to the selected fish species at the studied sites. Persistent exposure to untreated discharges from the Batts drain had potential health concerns as indicated by antioxidant system disruption and histological alterations in two economically important fish species, *O. niloticus* and *C. gariepinus*. The oxidative stress and structural damage in the liver and gills were attributed to the direct metallic injurious effects and excessive generation of ROS. *O. niloticus* showed better tolerance to the same level of pollution than *C. gariepinus* based on the level of lipid peroxidation and the frequency of histopathological changes. Continuous monitoring of the effluent quality near the Batts drain is needed to improve the health status of fish and increase their ability to survive and reproduce.

## Data Availability

The datasets used and/or analyzed during the current study are available from the corresponding author on reasonable request.
